# Peculiarities of situational and personal anxiety degree in the schoolchildren with ENT chronic diseases

**DOI:** 10.1186/s12955-017-0741-6

**Published:** 2017-08-25

**Authors:** Marine Mardiyan, Siranush Mkrtchyan, Artur Shukuryan, Armine Chopikyan, Razmik Dunamalyan, Lusine Danielyan

**Affiliations:** 1 Department of Health Governance and Economics, Yerevan State Medical University, 2 Koryun Street, 0025 Yerevan, Armenia; 2 Department of ENT diseases, Yerevan State Medical University, Yerevan, Armenia; 3 Division of Preventive Medicine, Yerevan State Medical University, Yerevan, Armenia

**Keywords:** Schoolchildren, ENT diseases, Quality of life

## Abstract

**Background:**

A number of the QL researches in case of different pathologies are being increased during the last decade. The existing traditional research methods provide mostly arbitrary data on the disease and its treatment, which are not sufficient for the schoolchildren overall psychological and social adaptation and wellness evaluation.

**Methods:**

The research object became schoolchildren of 3 randomly selected schools in Yerevan. 443 monitoring units formed the selection population. The degree of situational and personal anxiety was evaluated with the help of Spielberger’s and Gerbachevski’s tests.

**Results:**

According to our research data the anxiety degree was 29,2 ± 2,3 points among the girls and 12,5 ± 1,6 points among the boys, respectively. The individual anxiety level was especially high: it made up 44,5 ± 0,8 points, and that of the situational anxiety made up 37,2 ± 0,5 points (*p* < 0,05).

According to Gerbachovski’s test in the group of schoolchildren with ENT pathology those with a high level of demands made up 53,5 ± 3,2%, with a medium level of demands - 32,4 ± 3,0% and with a low level of demands −14,1 ± 2,2%. A number of the practically healthy schoolchildren with a low level of demands made up 50,3%, and with a high level – 30,7%.

**Conclusion:**

According to the investigation data those children who suffer from the ENT chronic diseases usually avoided communication, were sluggish and shy. According to the results of the research, the socio-psychological and adaptation abilities of children with the ENT chronic diseases were lower than those of the practically healthy (without ENT pathologies) coevals. This fact urges to improve the prophylactic measures provision in the mentioned pathologies aspect.

## Background

The disease influence on child’s psychology was continuously in the centre of many researchers’ attention [1, 2].

Chronic diseases affect children’s psychology and, probably because of that, very often outpatient service coveres only a small part of the existing problems [3–5].

Ongoing changes in the educational system often impact the children and teenagers health. Increased morbidity rate almost in all categories, the physical and mental development retardation and a high level of neuroticism were observed [6].

The physical and neuropsychological disorders are caused by frequently repeated chronic and severe ENT diseases. Physical development disorders, mental retardation, memory and cognition pathology and the delayed nervous reactions were typical of the children with ENT pathology [7].

This issue is of both medical and social background, since the upper respiratory infections lower the QL average score of the child and its family, which results in increase of socio-economic damages [8, 9].

The existing rehabilitation programs somehow help to improve the treatment results, ensure control of the disease and reduce its rate and severity [10]. However, these programs not always are potent to improve the quantitative dynamic characteristics in almost all spheres of the child’s activity. The existing traditional research methods provide just arbitrary data on the disease and its treatment. They don’t enable us to evaluate the schoolchildren’s psychological and social adaptation and wellness. Using the QL research methodology it becomes possible to carry out the overall analysis of the child’s physical, psychological and social activities, which is an onset of a new direction in interdisciplinary research development [11]. A number of the QL researches investigating in different pathologies influences increased during the last decade. The QL research provides very important indices comprising objective information on the productivity of programs implemented during the course of pathology or prophylaxis studies [12–15].

Hearing deterioration among children could sometimes remain unnoticeable and often cause disorders in mental development. Many researches results indicate to an existing connection between the chronic tonsillitis and neurological pathologies occurrence [6, 16–18].

A study of the psychological problems caused by chronic diseases is of great importance since the researches prove that availability of such diseases in childhood is a predisposing base for the further depressive disorders development [19], sometimes resulting in the suicide attempts [20].

## Methods

The research has been carried out in Yerevan, Armenia. The research organization program has been discussed and guaranteed by the Ethics Committee of the Yerevan State Medical University.

3 schools of Yerevan were randomly selected to be eligible for the research. 443 monitoring units formed a total cohort of selection with the following clustering:

1. 104 pupils – from the school after L. Tolstoy
2. 228 pupils – from the school after M. Heratsi
3. 111 pupils – from the school after L. Shant

The chronic ENT disease study in schoolchildren was performed by means of the specialized medical examinations. A monographic research of the investigated was carried out according to the principle of random selection. The selection distribution according to age-groups was as follows (Table 1): the 6–10 years group contained 111, the 11–14 years group – 104 and the15–18 years group - 228 children.

**Table 1:** Age and gender distribution of the selected schoolchildren

Age	Boys		Girls		Total	
	N	P ± m	N	P ± m	N	P ± m
6–10	60	29,7 ± 3,2	51	21,1 ± 2,6	111	23,5 ± 2,0
11–14	50	24,8 ± 3,0	54	22,3 ± 2,7	104	25,1 ± 2,1
15–17	92	45,5 ± 3,5	136	56,6 ± 3,2	228	51,4 ± 2,1
Total	202	100	241	100	443	100

### Procedure

The ENT disease study was carried out in the following stages:

The **1st stage** was a preliminary survey of parents. We developed a special form of questionnaire for the baseline data collection. The questionnaire included questions on the frequency of cases of severe ENT diseases, as well as of the chronic ENT pathology recurrence, allergic predisposition, heredity and dispensary control.

The survey was carried out openly. The parents’ survey results enabled us to obtain necessary information on the hereditary predisposition in the child’s medical history, complaints, dispensary control and the other chronic diseases, as well.

The **2nd stage** was the schoolchildren’s medical card data collection. Data on severe infectious diseases of the upper respiratory tract, recurrent tonsillitis, as well as dispensary control of the ENT diseases were collected.

The **3rd stage** was a clinical study of the ENT organs of schoolchildren by means of which the following methods of examination:

1. Anterior rhinoscopy and posterior rhinoscopy
2. Pharyngoscopy
3. Indirect laryngoscopy
4. Otoscopy

All the data were registered in the child’s survey card.

The research was carried out using the case-control design. According to the inclusion criteria an experimental group was formed using the casual randomized study method. The schoolchildren, who were not included in the project, formed the control group.

### Instruments

A degree of situational and personal anxiety has been evaluated by the Spielberger’s and Gerbachevski’s tests, the functional significance of which was as follows:

- The Spielberger’s test helped to evaluate the anxiety degree in schoolchildren with ENT pathology,
- The Gerbachevski’s test helped to evaluate the degree individual’s demand.

Spielberger’s test-questionnaire consists of 40 statements. Twenty judgments refer to determination of the situational anxiety level and the other twenty ones refer to determination of the personal anxiety level. To evaluate the anxiety level the evaluation key was applied. On the base of the respondent’s effective sensor the degrees of situational and personal anxieties were evaluated.

In practice the following scale of anxiety level evaluation is more applicable:

- ➢ up to 30 points – a low anxiety level
- ➢ from 31 to 44 points – a medium anxiety level
- ➢ 45 and higher – a high anxiety level

Gerbachevski’s test consisted of 42 statements. Among the problematic situations which occurred in the process of filling in the questionnaire the following types of them were noticeable: the cognitive, social, need of self-recognition, of self-estimation and other problems. On this base it was possible to evaluate an importance and a value of the problem, time and power realization and also the individual situation assessment. The test results showed the structural features of subject’s individuality. The following components of that stood out in the structure: personal motive, cognitive motive, motives of avoidance, competitiveness, activity modifications, self-respect, solving of the problem, evaluation of the assessed results score level, evaluation of one’s own potential, power systematization level, results general pattern and initiative. For each component the total score and transfer rules of the scores were calculated.

According to the Gerbachevski’s questionnaire, the degree of the individual’s demand was evaluated with the following points:

- ✓ 3–9 points – for a low level of the individual’s demand
- ✓ 10–16 points – for a medium level of the individual’s demand
- ✓ 17–21 points - for a high level of the individual’s demand.

The questionnaire enables us to reveal an individual’s psychological description, i. e. belonging to extro- or introverts, as well as the level of neuroticism typical of them. Two months prior to pretest two questionnaires were provided for 100 schoolchildren: for the Gerbachevsky’s test the Cronbach’s α was 0.85 and for the Spielberger test it was 0.87 which suggested certain corrections in the questionnaire to ensure clarity, wording and logic sequence.

## Data analysis

To analyze and evaluate the statistical material the following statistical methods of calculation of medium and relative indices, reliable evaluation by means of the Independent Sample Test, correlation analysis, binary logistic regression were applied.

The database was created by SPSS Statistics. The quanititative numbers describing the observation unit were converted to average arithmetic (M). For average veracity evaluation the arithmetic average error was calculated (m) and the veracity of average arithmetic was evaluated by Student’s *t* factor. Module *t* was equal to 2 (95% veracity *p* < 0,05). The binary logistic regression was applied to evaluate the connection between the features.

## Results

The studied population was represented by the boys, which made up 48.1% and the girls, which made up 51.9%, the majority of which (53.5%) were with the ENT diseases (Table 2).

**Table 2:** The comparative demographic and clinical characteristics of Armenian schoolchildren (with and without ENT pathologies)

Characteristic	Total cohort	Schoolchildren with ENT pathology	Schoolchildren without ENT pathology
N (%)	443	237 (53,5)	206 (46,5)
Boys, n (%)	202 (45.6)	112 (47.3)	90 (43.7)
Girls, n (%)	241 (54.4)	125 (52.7)	116(56.3)
Age Groups n (%)			
6–10 year	111 (25,0)	69 (29.1)	42 (20.4)
11–14 year	104 (23,5)	60 (25.3)	44 (21.4)
15–17 year	228 (51,5)	108 (45.6)	120 (58.2)

According to our research data got, the anxiety degree among the girls was 29,2 ± 2,3 points and among the boys it was 12,5 ± 1,6 points.

The individual anxiety level was especially more expressed: it made up 44,5 ± 0,8 points and that of situational anxiety was 37,2 ± 0,5 points (*p* < 0,05) (Table 3). The personal anxiety was of comparatively inert psychological character. The results showed that it was insignificantly changed.

**Table 3:** The comparative description of the medium level of personal and situational anxiety in the selected groups

Alarm parameters	Schoolchildren with ENT pathology			Schoolchildren without ENT pathology		
	M (95%)	σ	m	M (95%)	σ	m
Situational anxiety	37,2	4,02	0,54	17,84	4,7	1,7, *p* = 0,01
Personal anxiety	44,5	6,33	0,82	25,03	5,97	2,7, *p* = 0,01

Based on the situational anxiety level, we divided all the patients into 3 groups: of the situational anxiety of a low, a medium and a high levels (Table 4). It was revealed that 54.2% of schoolchildren with ENT diseases had a high level of situational anxiety, which was relatively higher as compared with the practically healthy schoolchildren (29.7%, *p* < 0,01).

**Table 4:** The comparative description of situational anxiety level in the selected groups

	Schoolchildren without ENT pathology	Schoolchildren with ENT pathology	*P* value
Level of situational anxiety^a^ (%)			
Low	57.3	5.3	<0.01
Medium	27.6	40.5	<0.01
High	29.7	54.2	<0.01

^a^Low level was up to 30 points; medium level − 31-44; high level - 45 and higher

According to the Gerbachovski’s test in the group of schoolchildren with ENT pathology, those with a high level of demands made up 53,5 ± 3,2%, the ones with a medium level of demands formed 32,4 ± 3,0% and those with a low level of demands were 14,1 ± 2,2%. A number of the practically healthy schoolchildren with a low level of demands made up 50,3%, and with a high level was 30,7% (Table 5).

**Table 5:** The comparative description of a “level of demands” by means of V.K. Gerbachovski’s questionnaire

Level of demands	Practically healthy schoolchildren		Schoolchildren with ENT pathology	
	n	P ± m	n	P ± m
Low −3-9 points	103	50,3 ± 3,5	33	14,1 ± 2,2
Medium - 10-16 points	39	19,0 ± 2,7	77	32,4 ± 3,0
High - 17-21 points	64	30,7 ± 3,2	127	53,5 ± 3,2
Total	206	100	237	100

**Fig. 1:**
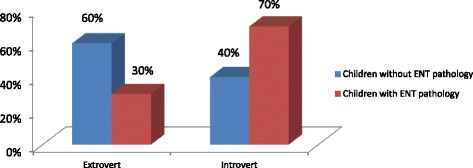
Extroverts and introverts in the 6–10 age group schoolchildren with and without ENT pathology (%)

According to psychological testing by means of the test-questionnaire, it was revealed that the majority of schoolchildren in the 6–10 age-groups without ENT pathology were extroverts (60%). Extroverts mostly were choleric personalities (80% choleric and 20% sanguine ones). Introverts mainly belonged to the group of children with ENT pathology (70%) and they mostly were melancholic (60%) and phlegmatic ones (40%) (Fig. 1).

**Fig. 2:**
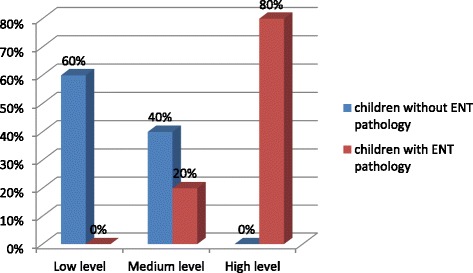
The level of neuroticism in the 6–10 age group schoolchildren with and without ENT pathology (%)

A high level of neuroticism was typical of 80% of children with ENT pathology in the 6–10 age-group, and a medium level of that - to 20%. A low level of neuroticism was typical of 60% children without ENT pathology and a medium level – of 40% (Fig. 2).

**Fig. 3:**
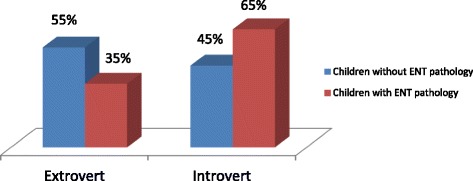
Extroverts and introverts in the 11–14 age group schoolchildren with and without ENT pathology (%)

The results of testing in the 11–14 age-group revealed that the majority of children with ENT pathology (65%) were introverts. Children without ENT pathology (55%) were extroverts. Introverts mainly belonged to the phlegmatic group (55%-phlegmatic, 45%-melancholic ones). Extroverts were mainly choleric personalities (70%-choleric, 30%-sanguine ones) (Fig. 3).

**Fig. 4:**
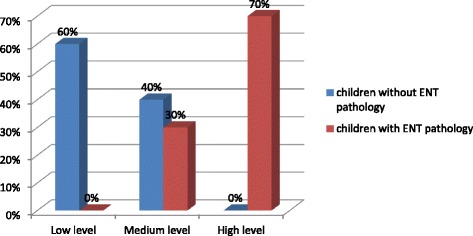
The level of neuroticism in the 11–14 age group schoolchildren with and without ENT pathology (%)

70% of schoolchildren with ENT pathology in the 11–14 age-group had a high level of neuroticism (low level of emotional stability), meanwhile 30% of them belonged to the medium level. 60% of schoolchildren without ENT pathology had a low level of neuroticism (high level of emotional stability) and 40% of them had a medium level of neuroticism (Fig. 4).

**Fig. 5:**
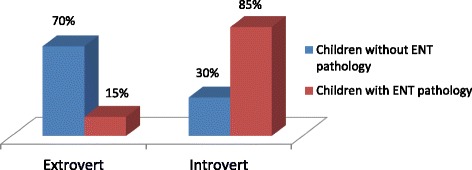
Extroverts and introverts in the 15–17 age group schoolchildren with and without ENT pathology (%)

Testing of schoolchildren in the 15–17 age-group revealed that the children with ENT pathology belonged to the group of introverts (85%). Introverts were mainly melancholic (phlegmatic - 43%, melancholic ones - 57%). A number of extroverts were high among the children without ENT pathology (70%). They were mainly choleric personalities (choleric ones - 69%, sanguine −31%) (Fig. 5).

**Fig. 6:**
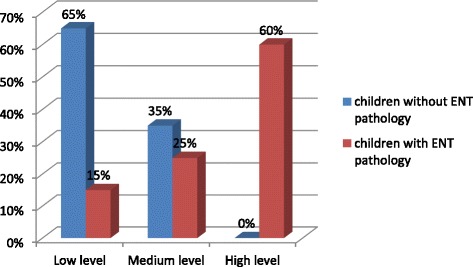
The level of neuroticism in the 15–17 age group schoolchildren with and without ENT pathology (%)

60% of the schoolchildren with ENT pathology in the 15–17 age-group had a high level of neuroticism (low level of emotional stability), 25% of them had a medium level and 15% of them - a low level of neuroticism – (high level of emotional stability). A low level of neuroticism was typical of 65% of children without ENT pathology (a high level of emotional stability) and the medium level was typical of 35% of them (Fig. 6).

## Discussion

This study underlines a fact of an influence of the chronic diseases onto the anxiety level in children of 6 to 17 years cohort. Anxiety disorders constitute the largest group of mental disorders in the most western societies and became the leading causes of disability [21].

While the problem of anxiety disorders is quite well-discussed in the accessible to us literature [22–25], anyway the studies devoted to an impact of the chronic diseases on anxiety level are rather rare [26–29].

According to our research data, the anxiety degree was relatively high among the girls as compared with the boys (*p* < 0,01), which was confirmed multiply by different researches on the psychological problems of males and females [30–38].

It should be noted that schoolchildren with the ENT pathology have a relatively high level of anxiety as compared with the group of healthy schoolchildren (*p* < 0,01) [39].

According to Spielberger’s test the personal anxiety degree is higher than the situational anxiety degree.

According to the research data the anxiety level increases with age (OR** = 0.27 *p* < 0.01, CI**:0.18–0.39) [40]. Connection between the personal anxiety and the expressiveness of ENT pathology is quite noticeable (OR = 0.44, *p* = 0.01, CI: 0.38–0.45).

According to the research data it was revealed that the ENT organ’s disfunction causes the communication disorders, which are of psychological importance.

We suggest that it is necessary to emphasize not only the schoolchildren with ENT dysfunctions with a high or a very high anxiety level, but also those who are too quiet. Much likely it is of a protective nature and could hinder full development of the individual. Thus, high scores on the scale are seemed to be a unique “call for help”, and vice versa, the extreme quietness can appear to be a quite dangerous signal about which the respondent doesn’t want to inform the experimenter.

It is necessary to increase the activity, provoke interest and the sense of responsibility. In order to make the research among schoolchildren more productive and comprehensive, to reveal their individual features, as well as aiming with the psychodiagnostic purposes, the children were given the psychological tests selected by psychologists.

According to our research data a low or a medium level of neuroticism was typical of the practically healthy children and its high level was mainly typical of the children with ENT pathology. This additionally confirms an assuredness that ENT diseases have psychological implications [7, 41].

## Conclusion

Overall, we can assume that in line with the test data, those children who suffer from the ENT chronic diseases usually avoid communication and are sluggish and shy, as well. According to the results of our research, the socio-psychological and adaptation abilities of the patients with ENT diseases were lower than those of the practically healthy coevals. This fact makes it necessary to improve the prophylactic measures.

Thus, in case of the ENT pathology it is necessary to examine the patient’s psychological status (personal and situational anxiety level) and to evaluate the boundary changes. In order to have an integrated approach to their rehabilitation, further examination of individual, psychological and social peculiarities of patients with ENT diseases are of crucial importance.

All the schoolchildren with ENT pathology were introverts regardless the age. They typically had a high level of neuroticism (a low level of emotional stability). The index was especially high in the 6–10 age group. While developing the preventive healthcare measures it is necessary to consider the child’s personal and situational levels of anxiety.

## Abbreviations

ENT: Ear, nose, throat
MES: Ministry of education and science
QL: Quality of life
RA: Republic of Armenia
